# Impact of combined use of intraoperative MRI and awake microsurgical resection on patients with gliomas: a systematic review and meta-analysis

**DOI:** 10.1007/s10143-021-01488-3

**Published:** 2021-02-03

**Authors:** Constantin Tuleasca, Henri-Arthur Leroy, Iulia Peciu-Florianu, Ondine Strachowski, Benoit Derre, Marc Levivier, Michael Schulder, Nicolas Reyns

**Affiliations:** 1 Neurosurgery and Neurooncology Service, Centre Hospitalier Regional Universitaire de Lille, Roger Salengro Hospital, Lille, France; 2 Department of Clinical Neurosciences, Neurosurgery Service and Gamma Knife Center, Lausanne University Hospital (CHUV), Lausanne, Switzerland; 3 Faculty of Biology and Medicine (FBM), University of Lausanne (Unil), Lausanne, Switzerland; 4 Signal Processing Laboratory (LTS 5), Ecole Polytechnique Fédérale de Lausanne (EPFL), Lausanne, Switzerland; 5 Department of Neurosurgery, Zucker School of Medicine at Hofstra/Northwell, Manhasset, NY USA

**Keywords:** Awake, Intraoperative MRI, Primary brain tumors, Resection, Complications

## Abstract

Microsurgical resection of primary brain tumors located within or near eloquent areas is challenging. Primary aim is to preserve neurological function, while maximizing the extent of resection (EOR), to optimize long-term neurooncological outcomes and quality of life. Here, we review the combined integration of awake craniotomy and intraoperative MRI (IoMRI) for primary brain tumors, due to their multiple challenges. A systematic review of the literature was performed, in accordance with the Prisma guidelines. Were included 13 series and a total number of 527 patients, who underwent 541 surgeries. We paid particular attention to operative time, rate of intraoperative seizures, rate of initial complete resection at the time of first IoMRI, the final complete gross total resection (GTR, complete radiological resection rates), and the immediate and definitive postoperative neurological complications. The mean duration of surgery was 6.3 h (median 7.05, range 3.8–7.9). The intraoperative seizure rate was 3.7% (range 1.4–6; I^2 = 0%, P heterogeneity = 0.569, standard error = 0.012, *p* = 0.002). The intraoperative complete resection rate at the time of first IoMRI was 35.2% (range 25.7–44.7; I^2 = 66.73%, P heterogeneity = 0.004, standard error = 0.048, *p* < 0.001). The rate of patients who underwent supplementary resection after one or several IoMRI was 46% (range 39.8–52.2; I^2 = 8.49%, P heterogeneity = 0.364, standard error = 0.032, *p* < 0.001). The GTR rate at discharge was 56.3% (range 47.5–65.1; I^2 = 60.19%, P heterogeneity = 0.01, standard error = 0.045, *p* < 0.001). The rate of immediate postoperative complications was 27.4% (range 15.2–39.6; I^2 = 92.62%, P heterogeneity < 0.001, standard error = 0.062, *p* < 0.001). The rate of permanent postoperative complications was 4.1% (range 1.3–6.9; I^2 = 38.52%, P heterogeneity = 0.123, standard error = 0.014, *p* = 0.004). Combined use of awake craniotomy and IoMRI can help in maximizing brain tumor resection in selected patients. The technical obstacles to doing so are not severe and can be managed by experienced neurosurgery and anesthesiology teams. The benefits of bringing these technologies to bear on patients with brain tumors in or near language areas are obvious. The lack of equipoise on this topic by experienced practitioners will make it difficult to do a prospective, randomized, clinical trial. In the opinion of the authors, such a trial would be unnecessary and would deprive some patients of the benefits of the best available methods for their tumor resections.

## Introduction

Surgical treatment of primary brain tumors located within or near eloquent areas is challenging. The primary goal is to preserve neurological function, while maximizing the extent of resection to optimize long-term neurooncological outcomes and thus quality of life.

In fact, it has been previously acknowledged that in primary brain tumors, the extent of resection (EOR) is associated with overall survival (OS) [3, 20, 44]. Moreover, increase in EOR reduces the incidence of tumor recurrence and further of malignant transformation in low-grade gliomas (LGG) [1, 27]. However, lesions in eloquent areas pose a particular challenge. Moreover, surgical resection is just a part of the multimodal management of patients with these lesions.

Reports of the use of intraoperative magnetic resonance imaging (IoMRI) have been conflicting with regard to an increase of the EOR [6, 16]. Intraoperative images were initially considered to lead to a 20% increase in the volume of total tumor resection [33, 48], especially for low-grade gliomas (LGG). However, in more recent series, this benefit might be considered much higher. The “flip side” benefit is in complication avoidance as a result of images that confirm that surgical goals have been reached. This is of particular importance for “eloquent” brain areas the injury to which can cause neurological deficits that will notably affect quality of life (QoL). The two surgical goals remain maximal EOR, while preserving neurological function [43].

The use of intraoperative electrical stimulation has become an accepted standard for defining cortical areas underlying eloquent function [2, 8, 34]. Use of this method has been shown to improve functional outcomes for surgical resection in close proximity to eloquent areas of cortical function [55].

Awake craniotomy has a long neurosurgical track record and is usually recommended for patients who need removal of lesions from the areas of eloquent brain where language function would be at risk, and in surgery for medically refractory epilepsy. The current goal of such surgery is to preserve neurological functions including motor, language, and cognitive, for patients with any type of lesions observed near or within eloquent areas of the brain [30].

Here, we sought to review the combined use and integration of the operative techniques of awake craniotomy and IoMRI [23]. Both pose multiple challenges and concerns with regard to multiple aspects, including patient selection, airway control during surgery, operative setup, physical constraints, prolonged operative time, costs, patient comfort, and safety issues.

## Methodology

**Fig. 1:**
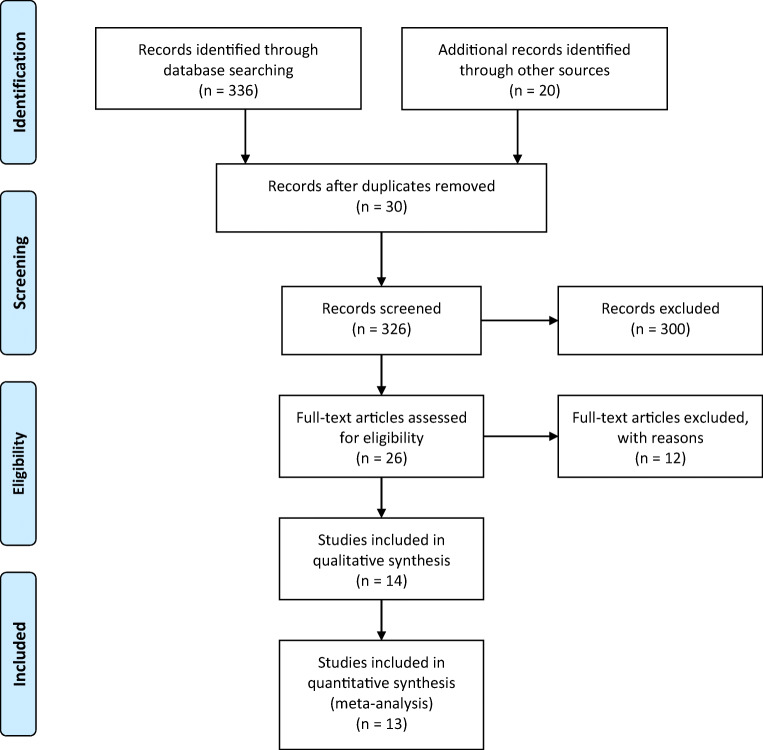
PRISMA flowchart

A PubMed search was performed for entries between January 1990 and February 2020 using the following query guidelines: ((awake AND (intraoperative OR intraoperative MRI, IoMRI)) AND (glioma)). We selected 1990 as a starting date to be sure we did not miss any relevant reference. Inclusion criteria required that each article be a peer-reviewed clinical study or case series of primary brain tumors treated with microsurgical resection, using both the IoMRI and the awake setting. As such, case reports, non-English studies, and conference papers or abstracts were not included. Exclusion criteria included studies reporting non-tumoral cases. The article selection is illustrated in Fig. 1, which included the studies reported further in Tables 1, 2, 3, and 4. Two separate reviewers applied the inclusion criteria to the PubMed search result; there were no disagreements. Moreover, four separate reviewers applied the exclusion criteria to the remaining articles. Were included 13 series and a total number of 527 patients, who underwent 541 surgeries.

**Table 1:** Basic demographic data

Series*	*N* pts, surgeries	Age (years) mean (range)	MRI (Tesla)	Left (L):right (R) hemisphere	Average operative time (h)	Average IoMRI time (min)	Intraoperative seizures
Nabavi et al. (2008)	34, 38		1.5	32:6	-	-	3/38 (7.9%)
Weingarten et al. (2009)	10, 10	25–57	1.5	-	6.8 h (3.8–8.7)	-	
Leuthardt et al. (2011)	12	32–60	1.5	9:3	7.9 (5.9–9.7)	45 (28.8–69)	
Lu et al. (2012)	30	45.5 (19–67)	3	-	-	-	
Tuominen et al. (2013)	20	44 (16–67)	0.23	-	4.45 (3.20–7.55)	-	
Maldaun et al. (2014)	41, 42	41.2 (22–70)	1.5	31:11	7.3 (4–13.9)	15.6 (5.1–27.1)	3/42 (7%)
Coburger et al. (2015)	26	-	-	-	-	-	-
Ghinda et al. (2016)	106	41.7 (18–76)	3				3/106 (2%) 1/106 (0.9%, generalized seizure)
Zhuang et al. (2016)	20	45 (27–67)	3				
Mehdorn et al. (2017)	106	-	-	-	-	-	-
Motomura et al. (2017)	25, 33	41 (28–67)	0.4	30:3	7.8 (4.4–12)		
White et al. (2018)	36		0.15		3.8 (2–6)	15	
Whiting et al. (2020)	61, 62	44.5 (19–78)		44:17			2/63 (3.2%)

*13 series including a total of 527 patients, who underwent 541 surgeries, *h* hours, *IoMRI* intraoperative MRI

**Table 2:** Function mapped, anatomical location, exact histology

Series	Function mapped	Anatomical location	Exact histology
Nabavi et al. (2008)	-	• L frontal operculum • L SMA • Dorsal temporal lobe	-
Weingarten et al. (2009)	• Language (*n*=5) • Motor (*n*=7) • Sensory (*n*=6)	• L temporal lobe (*n*=4) • Posterior L frontal (*n*=1) • Posterior R frontal (*n*=1) • L parietal (*n*=1) • R parietal (*n*=1)	-
Leuthardt et al. (2011)	• Language (*n*=3) • Motor (*n*=1) • Both (*n*=8)	• L frontal (*n*=14) • insula (*n*=8) • Parietal (*n*=4) • Temporal (*n*=4)	• Oligoastrocytoma 3/12 • Oligodendroglioma 2/12 • Anaplastic astrocytoma 1/12 • Anaplastic oligoastrocytoma 3/12 • Anaplastic oligodendroglioma 1/12 • Glioblastoma 3/12
Lu et al. (2012)	-	• Frontal (*n*=14) • Insular (*n*=8) • Parietal (*n*=4) • Temporal (*n*=4)	• Oligoastrocytoma 2/30 • Oligodendroglioma 4/30 • Diffuse astrocytoma 13/30 • Anaplastic oligodendroglioma 1/30 • Glioblastoma 10/30
Tuominen et al. (2013)	-	• Frontal (*n*=6) • Parietal (*n*=3) • Temporal (*n*=4) • More than one lobe (*n*=7)	• DNET 1/20 • Oligodendroglioma II (4/20), III (3/20) • Astrocytoma II (2/20), III (3/20) • Glioblastoma (4/20)
Maldaun et al. (2014)	• Language (*n*=9) • Speech and motor (*n*=21) • Motor (*n*=12)	• Frontal (*n*=17) • Parietal (*n*=4) • Temporal (*n*=5) • More than one lobe (*n*=9) • Insular (*n*=7)	• II and III (n=14) • IV (n=28)
Coburger et al. (2015)	• Language (*n*=26)	-	-
Ghinda et al. (2016)	-	• Frontal (*n*=48) • Parietal (*n*=9) • Temporal (*n*=18) • Insular (*n*=31)	• Oligoastrocytoma 2/30 • Oligodendroglioma 4/30 • Diffuse astrocytoma 13/30 • Anaplastic oligodendroglioma 1/30 • Glioblastoma 10/30
Mehdorn et al. (2017)	-	-	• Astrocytoma II 10/106 • Astrocytoma III 22/106 • Oligoastrocytoma 3/106 • Oligodendroglioma II 4/106 • Oligoastrocytoma III 5/106 • Anaplastic oligodendroglioma 6/106 • Glioblastoma 46/106 • Recurrent glioblastoma 6/106 • Gliosarcoma 2/106
Motomura et al. (2017)	-	• Frontal (*n*=15) • Parietal (*n*=5) • Temporal (*n*=1) • Insular (*n*=11) • Occipital (*n*=1)	• Astrocytoma II 9/33 • Oligodendroglioma II 2/33 • Oligoastrocytoma 7/33 • Anaplastic astrocytoma III 2/33 • Anaplastic oligodendroglioma 5/33 • Glioblastoma 4/33 • Pleomorphic xantoastrocytoma 1/33 • Gliosis 3/33
White et al. (2018)	• Purely language (22/36)	-	• Glioblastoma 17/36 • Astrocytoma 8/36 • Oligodendroglioma 7/36 • Ganglioglioma 1/36 • Mesial temporal sclerosis (1/36) • Cysticercosis (2/36)
Whiting et al. (2020)	• Speech alone 23/62 (37.1%) • Motor alone 24/62 (38.7%) • Both speech and motor 15/62 (24.2%)	• Frontal (*n*=34) • Temporal (*n*=7) • Parietal (*n*=9) • Frontal, extending adjacent lobes (*n*=6) • Temporal, extending to adjacent lobes (*n*=4) • Insular (*n*=1)	• II (28/63) • III (15/63) • IV (18/63)

**Table 3:** Pertinent data with regard to tumor resection

Series	Volumes Mean (range)	IoMRI complete resection	No further resection (*n*, percentage)	Further resection	Complete resection (details)	EOR (before and after IoMRI) Mean, range
Nabavi et al. (2008)	-	-	-	-	Yes	-
Weingarten et al. (2009)	-	1/10 (10%)	2/9 (22.2%)	7/9 (77.8%)	7/10 (70%) complete 3/10 (30%) to an eloquent margin	
Leuthardt et al. (2011)	-	7/12 (58.3%)	5/12 (41.7%)	6/12 (50%)	5/12 (41.7%) complete 2/12 (16.7%) nearly total 5/12 (41.7%) subtotal	-
Lu et al. (2012)	60 (8.4–216.7)	11/30 (36.7%)	19/30 (63.3%)	11/30 (36.7%)	18/30 (60%) complete (grace to IoMRI 7/18 (60%))	92.5% (75.1–97) 100% (92.6–100)
Tuominen et al. (2013)	-	-	-		10/20 (50%) complete	-
Maldaun et al. (2014)	49 (3.3.–154.2)	-	25/42 (59.5%)	17/42 (40.5%)	17/42 gross total (40.5%) (grace to IoMRI 7/17 (41%))	56% 67%
Coburger et al. (2015)	-	-	-		17/26 (65.4%)	-
Ghinda et al. (2016)	58 (3.5–181.3)	44/106 (41.5%)	32/62 (51.6%)	30/62 (48.4%)	64/106 (60.4%)	-
Zhuang et al. (2016)	-	16/30 (53%) GTR	-	-	23/30 (77%)	53% 77%
Mehdorn et al. (2017)	-	-	-	-	-	-
Motomura et al. (2017)	46.1 (0.6–196.4)	9/25 (36%)	9/16 (56.2%)	7/16 (43.8%)	-	-
White et al. (2018)	-	12/36 (33%)	16/24 (66.7%)	18/36 (50%)	-	-
Whiting et al. (2020)	-	14/62 (22.6%)	7/48 (14.6%)	41/48 (85.4%)	27/63 (42.8%)	-

**Table 4:** Postoperative neurological outcomes

Series	Immediate neurological complications	Persisting neurological complications	Neurological stability/improvement
Nabavi et al. (2008)	0/38 (0%)	-	-
Weingarten et al. (2009)	3/10 (30%)	-	-
Leuthardt et al. (2011)	5/12 (41.7%)	1/12 (8.3%)	11/12 (91.7%)
Lu et al. (2012)	12/30 (40%)	1/30 (3.3%)	-
Tuominen et al. (2013)	2/20 (10%)	1/20 (5%)	16/20 (80%)
Maldaun et al. (2014)	11/42 (26.2%)	1/42 (2.3%)	-
Coburger et al. (2015)	-	-	-
Ghinda et al. (2016)	48/106 (46.2%)	9/106 (8.7%)	-
Zhuang et al. (2016)	10/20 (50%)	1/18 (5.6%)	-
Mehdorn et al. (2017)	-	-	-
Motomura et al. (2017)	17/33 (51.5%)	4/33 (12%)	-
White et al. (2018)	3/36 (8.3%)	-	-
Whiting et al. (2020)	8/63 (12.7%)	0/63 (0%)	-

This study was performed in accordance with the published Preferred Reporting Items for Systematic Reviews and Meta-Analyses (PRISMA) guidelines [29].

We paid particular attention to operative time, rate of intraoperative seizures, rate of initial complete resection at the time of first IoMRI, the final complete resection rates at the time of last IoMRI, and the immediate and definitive postoperative neurological complications.

### Statistical analysis using OpenMeta (Analyst) and random-effects model

Due to the high variation in study characteristics, a statistical analysis using a binary random-effects model (DerSimonian-Laird method) was performed. We used OpenMeta (Analyst) from the Agency for Healthcare Research and Quality.

Weighted summary rates were determined using meta-analytical models. Testing for heterogeneity was performed for each meta-analysis.

Pooled estimates using meta-analytical techniques were obtained for all the outcomes previously described in the same section.

Case reports [36] or series not reporting the outcomes for all included patients were excluded [38].

## Results

### Number of studies and number of patients

Were included 13 series, encompassing a total number of 527 patients, who underwent 541 surgeries. All studies were retrospective cohort analysis.

The number of included patients ranged from 10 to 106.

Most commonly used MRI fields were 1.5 and 3 Tesla.

### Duration of surgery

**Fig. 2:**
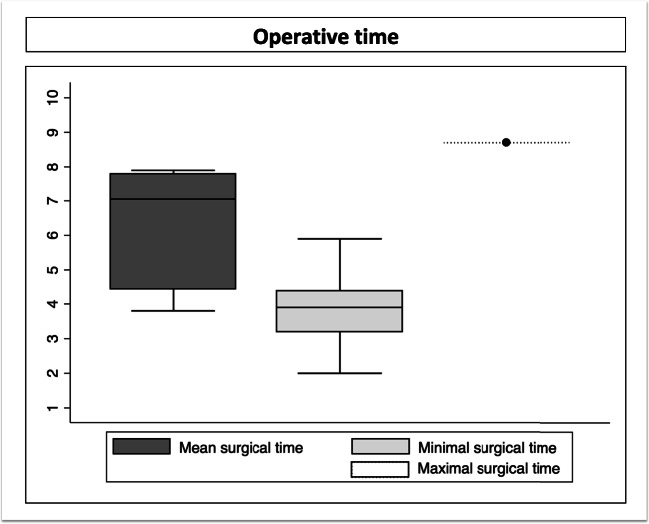
The mean, minimal, and maximal operative times (in hours)

The exact duration of surgery can be found in Table 1 and in Fig. 2. The mean duration of surgery was 6.3 hours (median 7.05, range 3.8-7.9). This has been reported on 6 studies, including 144 patients[22, 26, 30, 54, 56, 58].

### Intraoperative seizure rate

The intraoperative seizure rate was 3.7% (range 1.4–6; I^2 = 0%, P heterogeneity = 0.569, standard error = 0.012, *p* = 0.002). This has been reported in 4 studies, including 242 patients [12, 26, 31, 59]

### Complete resection at the time of first IoMRI

**Fig. 3:**
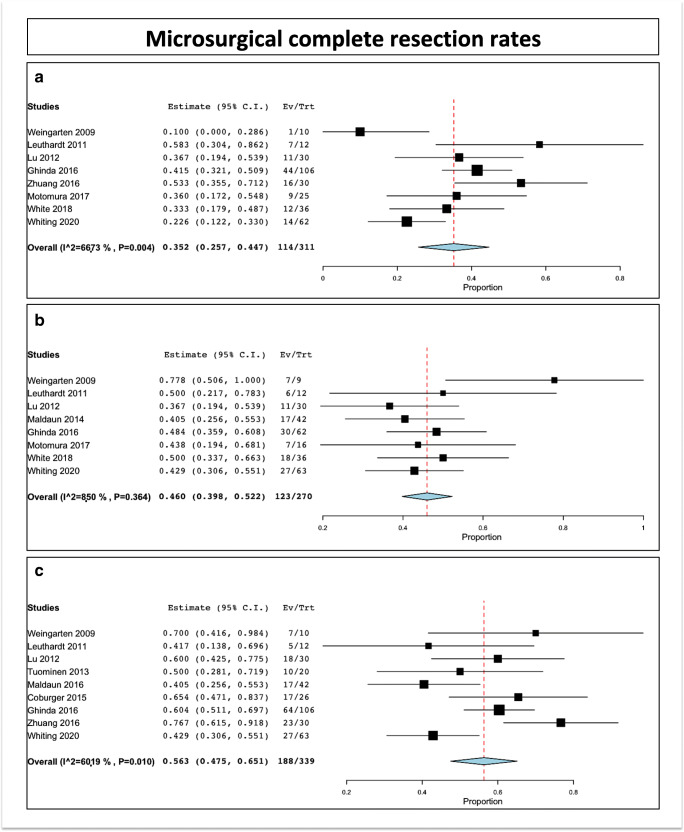
(A) Complete resection rates at the time of first IoMRI. (B) Patients benefitting from additional resection. (C) Final complete resection rates

The intraoperative GTR rate at the time of first IoMRI was 35.2% (range 25.7–44.7; I^2 = 66.73%, P heterogeneity = 0.004, standard error = 0.048, *p*< 0.001). This has been reported in 10 studies, including 303 patients (Fig. 3) [5, 12, 24, 26, 31, 54, 56, 59, 61].

### Patients who underwent complementary resection after IoMRI

The rate of patients who underwent supplementary resection after one or several IoMRI was 46% (range 39.8–52.2; I^2 = 8.49%, P heterogeneity = 0.364, standard error = 0.032, *p* < 0.001). This has been reported in 8 studies, including 255 patients [12, 22, 24, 26, 30, 56, 58, 59].

Moreover, the increment of complete resection after IoMRI was clearly reported in few studies [24, 26, 61].

### Gross total resection rates at discharge

The complete resection rate at discharge was 56.3% (range 47.5–65.1; I^2 = 60.19%, P heterogeneity = 0.01, standard error = 0.045, *p* < 0.001).

### Immediate neurological complications

**Fig. 4:**
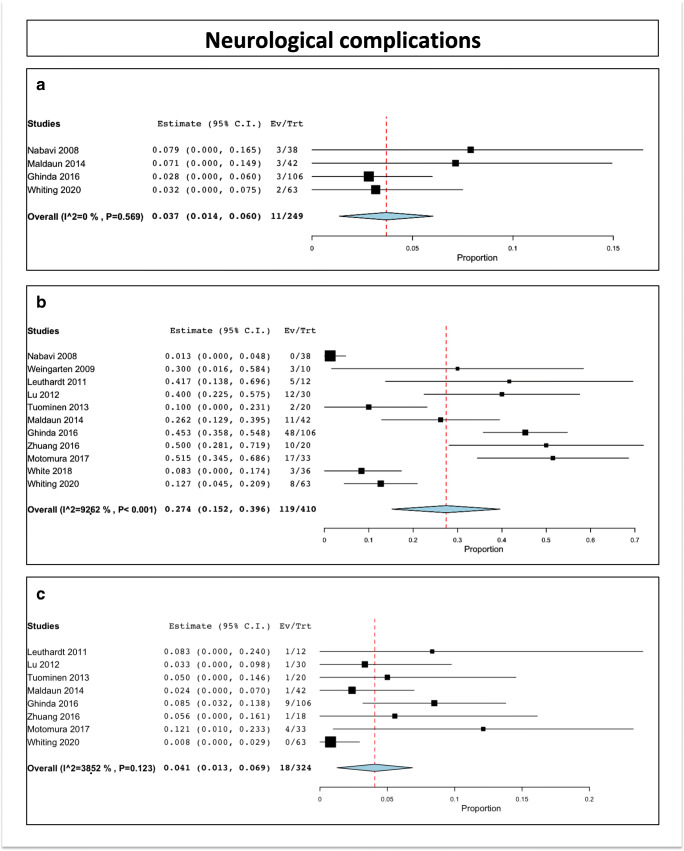
Neurological complications. (A) Intraoperative partial seizures. (B) Immediate postoperative complications. (C) Permanent postoperative complications

The rate of immediate postoperative complications was 27.4% (range 15.2–39.6; I^2 = 92.62%, P heterogeneity < 0.001, standard error = 0.062, *p* < 0.001). This has been reported in 11 studies, including 410 patients (Fig. 4) [12, 22, 24, 26, 30, 31, 54, 56, 58, 59, 61].

### Permanent neurological complications

The rate of permanent postoperative complications was 4.1% (range 1.3–6.9; I^2 = 38.52%, P heterogeneity = 0.123, standard error = 0.014, *p* = 0.004). This has been reported in 8 studies, including 324 patients [12, 22, 24, 26, 30, 54, 59, 61]

## Discussion

### Overall outcomes

In the present meta-analysis, we review the published literature on the use of awake microsurgical resection combined with intraoperative MRI. The mean intraoperative seizure rate was 3.7%. The mean initial (at the time of first IoMRI) and final (at the time of discharge) GT resection rates were 35.2% and 56.3%, respectively, including 46% of patients who had additional resection performed after intraoperative images showed residual tumor that could be safely resected.

The mean rate of immediate and permanent postoperative complications was 27.4% and 4.1%, respectively. Several aspects warrant for further interest and are detailed below.

#### Intraoperative MRI for glioma surgery and its benefits in general

It has been previously acknowledged that IoMRI increases the extent of resection and thus the overall survival in high-grade gliomas [49]. Moreover, for patients with low-grade gliomas, the use of IoMRI showed higher resection rates [15]. In this context, a recent and large multicentric trial showed that GT resection was an independent positive prognostic factor for PFS in low-grade gliomas and confirmed that IoMRI was significantly associated with GT resection [5]. In sum, intraoperative MRI data can be used to localize tumor remnants reliably and can also compensate for the effects of brain shift [32].

#### Intraoperative MRI and mapping

Currently, the cornerstone of treatment for low- and high-grade gliomas is maximal safe resection. Structural information related to tumor architecture as depicted by MRI studies should be completed, whenever necessary, with mapping modalities, determining the relationship of the tumor itself with the eloquent cortex and subcortical areas to avoid. The success of microsurgical resection for brain lesions close to eloquent cortex is linked to preservation and/or improvement of neurological functions and, thus, of the quality of life [11]. Integrating anatomical and functional data into the neuronavigation has nowadays become a standard of care in many departments dealing with neurooncology [25]. Moreover, advancement in neuroimaging and neurophysiological techniques and the appearance of the IoMRI have created a major paradigm shift and increased the EOR.

The main utility of the IoMRI is related to the compensation of brain shift and in intraoperative control of the microsurgical resection. Other imaging modalities can be further added, including functional MRI or diffusion tensor imaging, with the illustration of relevant tracts. The former is, however, limited by the radiological aspect of the intraoperative field, state of resection, etc. Combining IOM and IoMRI provides functional and structural information, which is extremely useful for the neurosurgeon, enabling higher resection rates, while preserving quality of life.

### Awake surgery contraindications

Awake surgery cannot be done in patients with pronounced aphasia, those who have minimal enough residual motor function, a score under 23 on the Mini Mental Status Examination, and those with apathic/disorganized behavior [31]. Other authors included also large body mass index and potentially difficult airway as reasons to rule out awake surgery [22].

### The major role of the anesthesiology team

The role of the anesthesiologist is crucial both preoperatively, to determine if the patient is a suitable candidate (both at medical and emotional levels, in terms of complete comfort during the procedure), and intraoperatively (patient able to talk and to follow commands [4]).

#### Patient position

The patient should be positioned in a way that she/he could have a large visual field view, including of the neuropsychologist’s team. Moreover, the patient should be able to visualize any images or numbers seen on the computer and further needing to be described.

The patient installation could also benefit from a transparent sterile field towards the neuropsychologists and anesthesiologists side, so as to facilitate the contact with both teams.

### The most common, the “AAA” technique

The most common procedure is a combination of general anesthesia and an awake technique, in what is known as the “asleep awake asleep” (AAA) technique. By this approach, the patient is placed under general anesthesia (without intubation) before and after brain tumor resection in awake. Usually, there is an initial phase of general anesthesia, followed by intraoperative awakening, and finally back to general anesthesia for the end of the surgery [40]. However, not all studies specifically detailed their awake technique. An important adjunct can be, in selected cases, intraoperative hypnosis [60].

### Intraoperative focal seizures

Cortical stimulation can provoke a seizure, the incidence of which is estimated to be between 5 and 20% [47]. These episodes usually can be managed by cold Ringer’s lactate solution irrigation of the cortex and patience, without the need for intravenous antiepileptic medication [46]. Postictal paresis might further limit the progress of surgery. Moreover, repeated seizures might lead to a long postictal period that can render the continuation of surgery difficult if not impossible. Of note, potential brain swelling can further complicate continuing the surgery. Hence, the interest of starting to progressively stimulate from lower to higher intensity.

### Cortical mapping

Penfield and Boldrey [37] first mapped the motor and sensory homunculi by stimulating the cerebral cortex with a monopolar electrode in conscious patients undergoing brain surgery for epilepsy. Bipolar ESM was later used during tumor surgery and for language area localization [57]. More recently, ESM has further been used to identify critical subcortical white matter tracts [10]. Motor mapping can be performed in patients under general anesthesia, with various results [42]. Of note, stronger cortical stimulation can favor ictal events; hence, the interest to couple it with EMG. However, speech mapping must be performed awake. Motor and speech testing are rehearsed before surgery. The most complex remains speech monitoring, ranging from naming to association and free narration. For speech localization during awake surgery, object naming is preferred to number counting. The heterogeneity of cortical areas activated during speech formation makes the cortical mapping of these areas in practice more demanding and time consuming compared to mapping motor areas [39].

Cortical mapping is thus a method that delinates functional areas during brain tumor surgery and preventing inadvertent injury to eloquent cortical and subcortical structures [57]. Many groups use the Ojemann bipolar cortical stimulator (Integra Inc). This probe has a 5-mm spacing between the electrodes. It relies on a constant current generator that produces a train of square-wave biphasic pulses. The most common settings include a 1 msec phase duration at a frequency of 60 Hz. For localization of primary language and motor cortices, stimulus is applied in increments starting at 1 mA. The Rolandic cortex may also be identified by somatosensory evoked potentials, to identify phase reversal and latency shift. No cortical site is stimulated twice in succession. Usually 2–3 mA is the maximum stimulus needed to localize the language center in an awake patient. In an asleep case, 10 mA may be needed to localize the motor cortex. If used correctly, a meta-analysis showed less than 4% rate of permanent severe neurological deficits in patients after resection with intraoperative stimulation mapping [7]. Of note, monopolar probe can be also safely used in an IoMRI setting both for navigation and stimulation purposes during the resection of primary brain tumors under general anesthesia [41]. Additionally, subcortical MEP mapping can be of further help to evaluate the distance towards the corticospinal tract during resection of motor-eloquent lesions thus reducing the risk of neurological deficit [50]. Combining information about intraoperative corticospinal tract together with direct electrical stimulation in the setting of IoMRI can enhance resection of brain tumors, with up to 77% having a GTR, as suggested by some authors [18].

### Language tasks of specific interest

Three language tasks should be performed: counting from 1 to a specific number (e.g., 50), picture naming, and word reading. One should also distinguish language deficits (speech arrest, anomia, or alexia) from dysarthria, which is caused by involuntary muscle (mouth or pharyngeal) contraction.

To localize language areas, fMRI and brain mapping using DCS have been commonly applied. Although fMRI is a noninvasive test, it has limited spatial resolution, with various sensitivity and specificity [13, 51]. The fMRI language activations do not necessarily correlate with intraoperative cortical stimulation findings. Moreover, because of the complexity of language area representation, fMRI has limited ability compared with cortical mapping for identification of such area; in fact, fMRI can be used for identifying language lateralization (left or right hemisphere), but less for precisely pointing out the cortical areas involved in language [53]. Therefore, DCS has been shown to be a reliable predictor of functional recovery [9, 45]

### Cortical resection

Cortical resection has to be performed at least 1–2 cm from areas of motor, sensory, or essential language function as identified by ESM [56]. However, more conservative recommendations suggest resection margins within 1–2 cm for speech function, and up to 0.5 cm for sensory and motor cortices. However, such conservative recommendation might preclude a maximal EOR. The neuropsychologist monitors the motor and speech function throughout the procedure. The authors increased the frequency of checks when the surgeons approached an identified area of eloquence. A resection, which is stopped if speech function deteriorates, can be resumed if clinical resolution resumes within 5 min.

### Extent of resection

In some of the series, there was no statistically significant difference in terms of EOR between low-grade and high-grade gliomas [26], while in others GTR was significantly lower in LGG as compared with HGG [12]. The usual strategy is to perform a staged volume surgery (with >= 1 IoMRI), while leaving in place a strategic tumor remnant, to avoid problems related to brain shift and further update neuronavigation [21] with IoMRI.

### Supramaximal resection and transient deficit

Some authors advocate performing a supramaximal resection despite the likelihood of a neurological deficit that hopefully will prove transient, because tissue plasticity and reorganization can further allow subsequent recovery [12]. In some of the studies, this was translated into a survival benefit [12]. However, the molecular biology of the tumors should be more taken into account in such type of studies

### Learning curve

Some of the authors stressed that there was a learning curve in applying these technologies [12]. This is considered related to the fact that surgeons often perform a less aggressive approach to decrease the rate of postoperative neurological deficits, which might result in a suboptimal tumor resection.

Lau et al. [19] further suggested that there is a learning curve associated with the ability to accurately assess intraoperative EOR during glioma surgery, and it may take more than a decade to become truly proficient. Moreover, the authors suggested that understanding the factors associated with the ability to accurately assess EOR will provide safer surgeries while maximizing tumor resection.

### Pitfalls and other adjuvant complementary approaches

Awake surgery with IoMRI takes longer. Thus, patients must be cooperative and motivated to participate with awake language mapping. Further refinement of the awake technique could be the patient’s sedation with Propofol. This has been reported by several centers [17, 35] reporting no adverse effects on the cortical stimulation after discontinuation of Propofol. Intraoperative fluorescence-guided resection has been shown to enhance the EOR [52]. One might consider that longer procedures might add to the risk of infection, deep venous thrombosis, and pulmonary embolism [56]. However, the complication rate did not increase [14].

A major aspect is the higher load of surgical information, which is demanding for the surgeons in terms of integration.

#### Sedation

Some of the authors [56] reported craniotomy being performed with local anesthesia and intravenous sedation; moreover, intravenous sedation was withdrawn after performing the craniotomy but before opening the dura mater. Other centers considered moderate intravenous sedation (Dexmedetomidine) and generous local anesthesia (bupivacaine) before patient positioning. Lu et al. [24] proposed moderate sedation with boluses of intravenous Propofol, while Tuominen et al. [54] used fentanyl and propofol (and further suspended sedation during cortical stimulation). In the series of Ghinda et al. [12], boluses of porpofol were used, followed by continuous administration of a low dose of remifentanil (0.01 microgrammes/Kg/min) or Dexmedetomidine (microgrammes/Kg/hour) during mapping.

#### Overall perspective

The combine setting of IoMRI and awake microsurgical resection is particularly useful for patients with tumors localized in functional areas. The complete postoperative resection rates might seem lower as classically reported for non-functional locations and the use of IoMRI. One useful strategy is to initially start resection after identifying the eloquent areas by cortical stimulation to optimize resection close to the area. IoMRI would become then useful to evaluate what one has left as residual tumor distant from such areas, to reassess neuronavigation data and optimize resection of this remnant.

### Comparative studies

A first comparative study was performed by Tuominen et al. [54], who compared patients having surgery with IoMRI and awake craniotomy to a group operated under general anesthesia and without cortical stimulation, while keeping the IoMRI setting. The authors concluded that awake craniotomy with bipolar cortical stimulation may help to reduce the risk of postoperative impairment following resection of tumors located in or near speech and motor areas.

A second comparative study by Mehdorn et al. [28] evaluated patients treated with two different approaches: awake craniotomy without (first period) and with (second period) intraoperative MRI. The authors suggested that there was a slight preponderance in redo surgeries for tumor remnant in the first period (11.2%) as compared with the second (7.4%). Moreover, the intervals between surgeries in both groups depended mainly on the histological grade. An interesting aspect was that patients with low-grade gliomas in the second series did not experience recurrences as frequently as those in the first series.

#### Major limitation in the current studies

One of the major limitations, which should be addressed by further studies, is to correctly report the increment of complete resections from first to last IoMRI. This would better enhance the utility of such an approach and provide the reader a correct information.

A second limitation is related to the awake examination after IoMRI. Moreover, further studies should report the exact number of IoMRI assessments, but also whether or not the patients have been putted into sleep or not during these single/multiple examinations.

## Conclusion

Awake craniotomy with language mapping can be combined successfully with IoMRI to maximize resection of brain tumors in selected patients and preserving neurological function. The technical obstacles to doing so are not severe and can be managed by experienced neurosurgery and anesthesiology teams. The benefits of bringing these technologies to bear on patients with brain tumors in or near language areas are obvious. The lack of equipoise on this topic by experienced practitioners will make it difficult to do a prospective, randomized, clinical trial.

## Data Availability

Data is presented in tables and figures, as it is a systematic review.

## Code availability

N/A
